# Combination therapy of renal cell carcinoma or breast cancer patients with dendritic cell vaccine and IL-2: results from a phase I/II trial

**DOI:** 10.1186/1479-5876-9-178

**Published:** 2011-10-20

**Authors:** Soyoung Baek, Choung-Soo Kim, Sung-Bae Kim, Yong-man Kim, Seog-Woon Kwon, YongMan Kim, HyunSoo Kim, Hyunah Lee

**Affiliations:** 1 Office of Biomedical Research, Samsung Medical Center, Sungkyunkwan University School of Medicine, Seoul, KOREA; 2 Dept. Urology, Asan Medical Center, University of Ulsan College of Medicine, Seoul, Korea; 3 Dept. Oncology, Asan Medical Center, University of Ulsan College of Medicine, Seoul, Korea; 4 Dept. Gynecology, Asan Medical Center, University of Ulsan College of Medicine, Seoul, Korea; 5 Dept. Laboratory Medicine, Asan Medical Center, University of Ulsan College of Medicine, Seoul, Korea; 6 FCB-Pharmicell Co.Ltd, Seoul, Korea

**Keywords:** Dendritic cell vaccine, Renal cell carcinoma, Breast cancer, Phase I/II trial, Immune response

## Abstract

**Background:**

Ten cancer patients (Six renal cell carcinoma and four breast cancer patients) were treated in a phase I/II study with a vaccine composed of autologous dendritic cells (DCs) and IL-2 to evaluate the DC vaccine-related toxicity and antigen-specific immune alteration.

**Methods:**

Cancer patients were treated twice with autologous CD34^+ ^hematopoietic stem cell-derived, GM-CSF/IFN-γ-differentiated DCs pulsed with autologous tumor lysate and KLH, by 4-week interval. Following each subcutaneous injection of therapeutic DCs, low-dose (200 MIU) IL-2 was introduced for 14 consecutive days as an immune adjuvant. To determine the DC vaccine-induced immunological alterations, the KLH-specific lymphocyte proliferation, number of IFN-γ secreting T cells (ELISPOT assay), NK activity and the cytokine modulation were measured.

**Results:**

Cultured-DCs expressing HLA-DR, CD11c, CD83, and B7.1/B7.2 produced IL-12p70. After vaccination, the patients tolerated it. Clinical response was observed in one RCC patient as stable disease. However DC-vaccine related antigen-specific immune responses including peripheral blood lymphocyte proliferation and the number of IFN-r secreting cells were induced in six patients without clear correlation with clinical responses. Also NK activity was induced significantly in six patients after vaccination. DC vaccine-related decrease of TGF-β level or increase of IL-12p70 level and decline of CD4^+^CD25^+ ^T cells were observed in three patients. However only in the RCC patient whose disease stabilized, combination of stimulatory as well as inhibitory immune alterations including induction of IFN-γ secreting T cell with reduction of CD4^+ ^CD25^+ ^T cell were correlated with clinical responses.

**Conclusion:**

Data indicated that DC vaccine combined with IL-2 is well tolerated without major side effects. DC vaccine induced the specific immunity against introduced antigen. Combinatorial alterations of immunological parameters indicating antigen-specific immune induction along with reduction of inhibitory immunity were correlated with clinical responses in DC vaccine treated patients.

## Background

As a professional antigen-presenting cell (APCs), DC induces antigen specific cytotoxic T lymphocyte (CTL) response thus DC vaccine has been anticipated as a cancer treatment regimen [1,2]. Subsets of therapeutic-DCs are known to have functional importance. Tumor antigen-specific immunity is induced by myeloid-DCs cultured from peripheral monocytes or hematopoietic stem cells (HSC). Cultured therapeutic myeloid-DCs induce not only the antigen-specific effecter T cells (both Th and CTLs) but also NK cell activity which can help the induction of anti-tumor responses [3-6]. Reciprocally, NK cells can activate DCs, enhancing their ability to produce pro-inflammatory cytokines and stimulate T helper and cytotoxic T lymphocyte responses of tumor-specific CD4^+ ^and CD8^+ ^T cells [4,5,7,8].

Renal cell carcinoma (RCC) accounts for 2-3% of all adult cancers. Although surgery is the primary curative therapy for patients with localized RCC, the prognosis for patients with advanced metastatic disease is poor, with a 5-yr survival rate of < 10% [9]. Because RCC is one of the most immune responsive cancers in human, immune- therapy with tumor infiltrating lymphocytes or lymphokine-activated killer cell has been studied with considerable side effects [10,11]. More recently, emphasis has shifted to the use of DCs to actively immunize cancer patients with their own DCs loaded with tumor antigens [12-16]. Unlike RCC, breast cancer is traditionally considered as a poorly immunogenic tumor, there are evidences for DC recruitment within tumor-microenvironment. Reports indicated the favorable impacts of intra-tumoral activated DCs on breast cancer patients' survival [17-19]. Furthermore, there is a striking paucity of activated DCs within the primary draining or sentinel lymph nodes of breast cancers [20,21]. In this study, the significance of the DC vaccine combined with IL-2 in renal cell carcinoma and breast cancer patients is presented regarding the relevance between the clinical and immunological responses.

## Materials and methods

### Culture Media and Reagents

Complete medium (CM) including X-VIVO 20 (BioWhittaker, Walkersville, MD, USA) supplemented with 1% human albumin (Green Cross, Seoul, South Korea), 2 mM glutamine, and 100 U/mL penicillin plus 100 μg/mL streptomycin (Gibco, Grand Island, NY, USA) were used to culture therapeutic-DCs. Recombinant human GM-CSF (LG Life Sciences, Seoul, South Korea), IFN-γ (R&D Systems, Minneapolis, MN, USA) were used to derive DCs. ELISA detection of IFN-γ, IL-10, and IL-12p70 release in therapeutic-DC culture supernatant was performed using commercially available ELISA kits (R&D Systems, Minneapolis, MN, USA). Detection of IL-10, IL-12, TGF-β and IFN-γ release in patient's plasma was performed using ELISA kits OptEIA™ (E-bioscience, San Jose, CA, USA) and Quantikine^® ^(R&D Systems Inc., Minneapolis, MN, USA) for VEGF. Keyhole limpet hemocyanin (KLH; Calbiochem, Germany) were used adjuvant. K562 is a human leukemia cell line for NK activity that was purchased from the American Type Culture Collection (ATCC, Rockville, MD, USA).

### Patients' Criteria

The clinical trial protocol for this study was approved by the Institutional Review Board of the Asan Medical Center on 2004. Korean Food and Drug Administration (KFDA) approved the protocol as an Investigative Trial on 2004. Patients were informed of the investigative nature of this study, and written consent in accordance with institutional regulations was obtained prior to study entry. Patients with five stage IV and one recurrent stage II renal cell carcinoma or stage IIb~IV breast cancer were enrolled for this study. Patients were enrolled from Jan. 2005 to Jan. 2007 (Table 1). The median age was 48 years (range, 24-67 years).

**Table 1:** Patient's characteristics

Pt. #	Age /sex	Stage /Grade	Diagnosis	Metastasis
R-M-1	62/M	TxNxM1(IV)	Clear cell RCC	Lung, Rib
R-G-2	56/M	T3bN1M1(IV)	Clear cell RCC	Lung, Paratracheal LN
R-K-3	67/M	T2N0M1(IV)	Clear cell RCC	Lung, Brain, Contralateral Kidney,
R-H-4	29/M	TxN2M0(II)	RCC	Local Recurrence
R-N-5	24/F	T2N2M1(IV)	Papillary RCC	Lung, Lower Neck, Supraclavicular & Retroperitoneal LN, Local Recurrence
R-L-6	62/M	TxNxM1(IV)	RCC	Thorax, Lung, Mediastinum
B-S-1	43/F	IV	Lt.breast cancer	Breast, Lung
B-H-2	29/F	IV	Bilateral breast cancer	Breast, Lung, skin, pleural
B-L-3	59/F	IIIC	Brest cancer	LN, chest wall, bone meta
B-Y-4	66/F	IIB	Rt.breast cancer	Bone, pleural liver

### Generation of Therapeutic-DCs

Therapeutic-DC was prepared in the GMP facility at the FCB-Pharmicell, Korea. To generate hematopoietic stem cell (HSC)-derived DC, CD34^+ ^cells were purified from the washed PBMCs by MACS™ cell separation system using anti-CD34-coated magnetic beads (Miltenyi Biotec, Germany). Enriched CD34^+ ^cells were resuspended in CM supplemented with GM-CSF (50 ng/mL), plated at a density of 2~3 × 10^5^/cm^2 ^in 75- cm^2 ^flasks. On day 7, culture medium was replaced with CM containing GM-CSF (50 ng/mL) and IFN-γ (10 ng/mL), and the cells were cultured for another 7 days. On day 13, autologous tumor lysate (30 μg/mL) and 1 μg/mL of KLH were pulsed to the cells for overnight. To make tumor lysate, resected tumors were rinsed with PBS and subsequently lysed by 5 cycles of freezing in liquid nitrogen and thawing in 37°C water bath. The tumor lysate was centrifuged, and the supernatant was filter-sterilized and stored at -80°C until use. Protein concentrations of the tumor lysate were determined using BCA Protein Assay Reagent Kit (Pierce, Rockford, IL, USA).

On day 14 of culture, the DCs were harvested, washed, and resuspended to a final concentration of 1 × 10^7 ^cells per mL in saline solution for the injection. Criteria for release of clinical grade DCs included viability greater than 70% and absence of microbial contamination (bacteria, fungus, and mycoplasma).

### Vaccination

**Figure 1:**
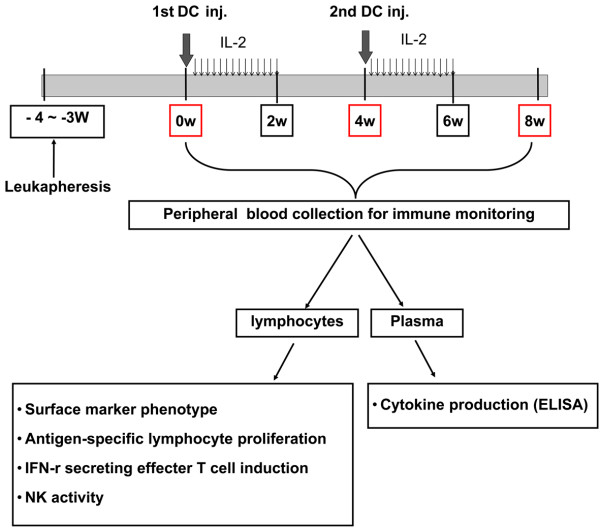
**Vaccination and immune monitoring Protocol**.

Autologous DCs (5 × 10^7^/5 mL) were injected subcutaneously adjacent to axillary lymph node. Six to ten hours after DC injection, IL-2 (2 × 10^6 ^IU; Novartis, Basel, Switzerland) administration began into an abdomen subcutaneously for consecutive 14 days. Patients were re-vaccinated 4 weeks after the first DC injection. Responses were analyzed after 2 cycles of DC vaccine and IL-2 treatment. Immune responses were evaluated at 0, 4 and 8 weeks of first DC treatment (Figure 1).

### Therapeutic-DC Characterization

#### Immunophenotypic analysis by flow cytometry

Phenotype of cultured cells was analyzed by direct immunofluorescence staining of cell surface antigens using FITC or PE conjugated antibodies against CD1a, CD40, CD11c, CD80, CD83, CD86, HLA-DR, and the appropriate isotype matched controls (BD Bioscience Pharmingen, San Diego, CA, USA). Samples were analyzed on EPICS XL-MCL flow cytometer (Beckman Coulter, USA) using Expo32 ADC analysis software.

#### Cytokine secretion

Culture supernatants from day 7 (GM-CSF) and day 14 (GM-CSF+IFN-γ) were obtained to measure the cytokine secreted from the cells. To characterize the type of differentiated DCs, the level of IL-12 and IL-10 along with IFN-γ was observed by ELISA kit (R&D Systems Inc., Minneapolis, MN, USA).

### Immune Monitoring

#### NK activity

Lymphocytes from the patient's peripheral blood served as effector cells (E). Log-phase K562 cells, a human chronic myelogenous leukemia cell line sensitive to NK-cell killing, were labeled with Na_2_^51^CrO_4 _and used as target cells (T). Labeled K562 cells (1 × 10^4 ^cells) and the selected concentration of effector cells (E: T = 25:1 or 50:1) were dispensed in a U-bottomed 96-well micro titer plate. After 4 hour incubation at 37°C in a humidified 5% CO_2 _incubator, the supernatant was removed and the radioactivity levels were assessed in a Wallac 1470 Wizard™ gamma counter (Finland). Spontaneous release (SR) and total release (TR) were measured in the supernatants of target cells incubated with either culture medium or 1N HCL. NK-cell activity was calculated by following equation;

NK cell activity (%)=[(experimental cpm−SR cpm)÷(TR cpm−SR cpm)]×100

#### Phenotype analysis

Fluorescence (either FITC or PE)-tagged antibodies were incubated with lymphocytes for 40 min at 4°C and the fluorescence was detected with FACSVINTAGE (Becton-Dickson, Mountain View, CA) within 2 hrs after antibody staining. Following antibodies were utilized: CD16/CD56 for NK cell subsets and CD4/CD25 for the T cell subset (BD Pharmingen, San Diego, CA, USA). Mouse IgG1-FITC and mouse IgG2a-RPE (DAKO, Glostrup, Denmark) were used as negative control.

#### Lymphocyte proliferation assay

Peripheral blood samples were collected in the heparin tube before and after 8 weeks of DC vaccination. Lymphocytes were separated by density gradient centrifugation using lymphocyte separation medium^® ^(Cellgro, Mediatech, USA). Cells were seeded in triplicates in 96-well plates (2 × 10^5 ^cells/well) with KLH (10 μg/ml) at 37°C, in humidified and 5% CO_2_-conditioned air for 4 days. Cells were loaded with 1 μCi^3^H-thymidine/well (Perkin-Elmer Inc., MA, USA) during the last 18 h of incubation. Cells were harvested on the glass micro-fiber filter using a PhD^® ^cell harvester (Cambridge Technology Inc., Cambridge, MA, USA). The proliferative response was determined by^3^H-thymidine incorporation using a liquid scintillation counter (Beckman LS 6500; Beckman Instruments Inc., Fullerton, CA, USA).

#### Analysis of IFN-γ producing cells using ELISPOT assay

The ELISPOT assay was adopted to detect and enumerate individual cells that secrete IFN-γ protein *in vitro *upon exposure to antigen. ELISPOT assays were performed according to the manufacturer's instruction (AID, Strassberg, Germany). In brief, using magnetic bead cell separation system (MACS™ Miltenyi Biotec, Germany) CD3^+ ^T cells were purified from the patient's peripheral lymphocytes (2 × 10^5 ^cells) and stimulated *in vitro *with KLH (10 μg/ml) on a 96-well pre-coated plate. The plate was incubated for 20 h at 37°C with 5% CO_2_. After washing, each well was added with detection antibody and was incubated for 2 h at room temperature. The plate was incubated with alkaline phosphatase conjugate and developed with BCIP/NBT substrate solution. Visible spots were enumerated using an automated AID ELISPOT reader (AID, Strassberg, Germany) and the default program.

#### Measurement of cytokine level

Alterations in blood circulating concentrations of IL-10, IL-12, TGF-β, VEGF and IFN-γ during immunotherapy were measured by ELISA. Plasma was obtained from the patient's peripheral blood before, during, or after treatment and stored at -70°C until the ELISA was performed using commercial kits; OptEIA™ (E-bioscience, San Jose, CA, USA) for IL-10, IL-12, TGF-β, IFN-γ and Quantikine^® ^(R&D Systems Inc., Minneapolis, MN, USA) for VEGF.

### Statistical Analysis

Statistical significance was determined using student's T-test. A value of *p *< 0.05 was considered significant.

## Results

### Therapeutic-DC Generation from the Autologous HSCs

**Figure 2:**
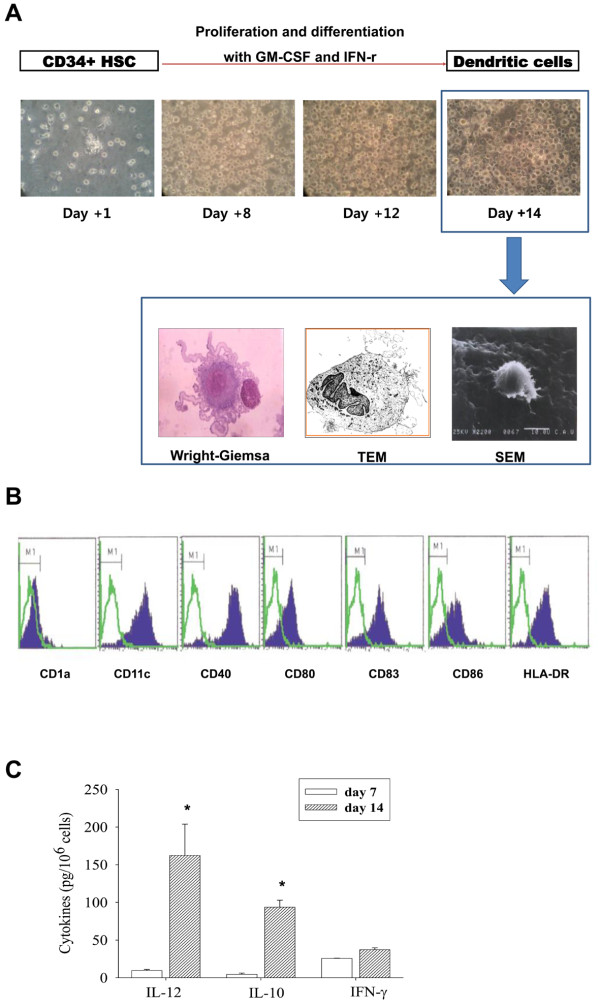
**Therapeutic-DC Generation from the Autologous HSCs**. DCs were differentiated from the patient's CD34^+ ^hematopoietic stem cells as described in materials and methods. (A) Differentiated cells on day 14 have many dendrites on the surface. Scant intracellular organelles are observed by Wright Giemsa stain × 1000, Transmission electronic microscope (TEM) and Scanning electron microscope (SEM) × 2200. (B) Phenotype alterations of day 14 cultured cells were analyzed by flow cytometry (Green line: control, Blue solid line: cultured cells). The cultured cells expressed mature myeloid-DC markers including CD11c and CD83. (C) Cytokines secreted into the culture supernatant (day 7 and day 14 after HSC culture setting) were measured by ELISA. Amount of cytokines were expressed as pg/10^6 ^cultured cells. Stars indicate the statistical significance comparing day 7 vs. day 14 cytokine secretion by *p *< 0.05.

Therapeutic-DC culture started with purified CD34^+ ^HSCs from patient's own PBMC. Magnetic bead purified cells were mixture of CD34^+ ^HSCs (63.03 ± 11.35%) and CD14^+ ^monocytes (21.10 ± 5.38%) (data not shown). Following culture of purified cells with GM-CSF and IFN-γ, cells become proliferated and differentiated into DCs as observed with microscopes (inverted as well as electron microscope: Figure 2A). On day 14 after culture, cells were expanded about 3000% of initiating HSCs with DC phenotype including CD11c^++^, CD40^+++^, CD54^++^, CD80^+^, CD86^+^, HLA class I^++ ^and HLA class II^++^. The maturation state of the cultured DCs was confirmed by the expression of CD83^++ ^with disappearing CD1a expression (Figure 2B). Reduction of CD14 expression in the day 14 cultured cells (from 21.10 ± 5.38% to 1.97 ± 0.29%) also verified the DC differentiation (data not shown). Day 14 cultured DC produced higher amount of IL-12 than IL-10 which allow to expecting the induction of stronger Th1 cellular immunity desirable for cancer vaccine (Figure 2C).

### Anti-tumor Response to Therapeutic DC with IL-2 Combination Therapy

Six patients with renal cell carcinoma and four patients with breast cancer were treated with 5 × 10^7 ^DCs and 14 consecutive IL-2 injections in a single vaccination protocol, and all patients received two vaccinations at a one-month interval (Figure 1). No adverse effects inhibiting the vaccination were observed. By completion of treatment, one case of renal cell carcinoma showed stable disease and 9 cases showed progression of disease (Table 2).

**Table 2:** Response to DC vaccination and results of immune monitoring

Pt. #	DC dose	**Total No**. vaccines	Response	Adverse effects	Proliferation assay	NK activity	IFN-r ELISPOT	CD4+25+cell proportion
R-M-1	5 × 10^7^	2	PD	No	↑↑	↑	-	↓
R-G-2	5 × 10^7^	2	SD	No	↑↑	↑	↑	↓
R-K-3	5 × 10^7^	3	PD	No	-	-	↑	↑
R-H-4	5 × 10^7^	2	PD	No	-	↑	-	-
R-N-5	5 × 10^7^	2	PD	No	-	↑	-	-
R-L-6	5 × 10^7^	2	PD	No	-	-	↓	-
B-S-1	5 × 10^7^	2	PD	No	↑	↑	↓	↑
B-H-2	5 × 10^7^	2	PD	No	↑	↑	-	↑
B-L-3	5 × 10^7^	2	PD	No	↑	-	↓	↑
B-Y-4	5 × 10^7^	2	PD	No	-	↓	-	-

↑↑: Highly significant (*p *< 0.001) increased ↑: Significant (*p *< 0.05) increased

↓: Significant (*p *< 0.05) decreased -: No significant change

### NK Activity and Cytotoxic NK Cell Proportion

**Figure 3:**
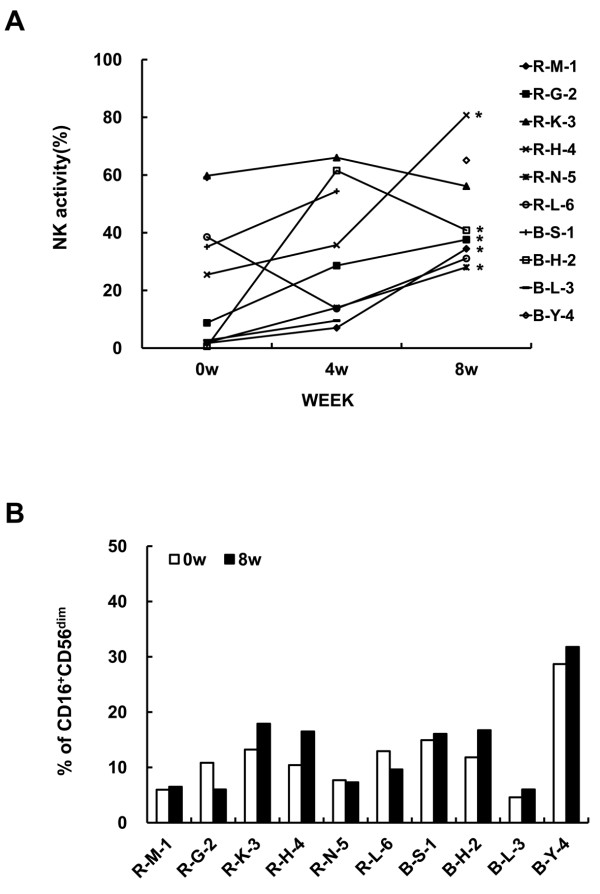
**NK Activity and Cytotoxic NK Cell Proportion**. (A) NK activity was measured as described in materials and methods using PBMC (effector) obtained at day 0 (baseline), during (4W) and after the vaccination (8W). Stars indicate the statistical significance comparing day 0 vs. after the vaccination by *p *< 0.05. (B) Alteration of NK cell (0W: White bar) and after the vaccination (8W: Black bar) was measured by flow cytometry. Numbers indicate the CD16-FITC^+ ^and CD56-PE^dim ^cytotoxic NK cell proportion.

To determine the influence of DC vaccination on NK activity in the patients, peripheral blood lymphocytes were collected before (w0), during (w4) and after vaccination (w8). NK activity of six patients (R-M-1, R-G-2, R-H-4, R-N-5, B-S-1 and B-H-2) increased after the 2^nd ^vaccination at w8 observation (Figure 3A). In these patients, slight modulation or maintenance of cytotoxic NK cell proportion (CD16^+^CD56^dim^) was observed. A correlation between the proportion and activity of cytotoxic NK cells was observed in only two patients (R-H-4, B-H-2) (Figure 3B). And reduction of cytotoxic NK cell proportion was observed in one patient with increased NK activity (R-G-2).

### Antigen-Specific Lymphocyte Proliferation

**Figure 4:**
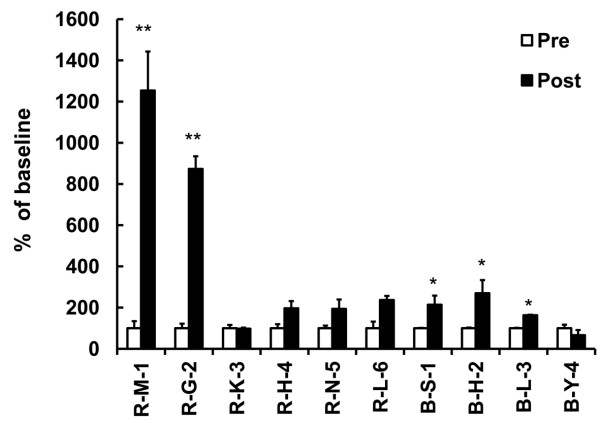
**Antigen-Specific Lymphocyte Proliferation**. The proliferative response was determined by^3^H-thymidine incorporation (DPM). Data plotted as the relative change from pre-vaccination baseline. Stars are indicated the statistical significance (*p < 0.05; **p < 0.001) comparing pre (White bar) vs. post-vaccination (Black bar).

A proliferation assay was performed with peripheral blood lymphocytes taken from the patients before and after the 2^nd ^vaccination cycle. Cells were stimulated *in vitro *with KLH, which was pulsed into the therapeutic DCs as a surrogate antigen. In two patients (R-M-1, R-G-2), the proliferative response was significantly increased (*P *< 0.001) after vaccination (Figure 4) and the proportion of CD4^+^CD25^+ ^cell was reduced, concomitantly (from 12.47% to 1.7% and from 10.0% to 1.59% for R-M-1 and R-G-2, respectively; Table 3).

**Table 3:** Percentage of CD4^+^25^+ ^T cell Subset

Pt. #	Pre(0w)	Post(8w)
R-M-1	12.47%	1.70%
R-G-2	10.00%	1.59%
R-K-3	1.02%	1.88%
R-H-4	4.40%	5.83%
R-N-5	4.74%	6.20%
R-L-6	2.70%	3.59%
B-S-1	3.31%	5.94%
B-H-2	4.54%	9.29%
B-L-3	3.66%	7.05%
B-Y-4	8.18%	6.08%

### IFN-γ Secreting T Cells

**Figure 5:**
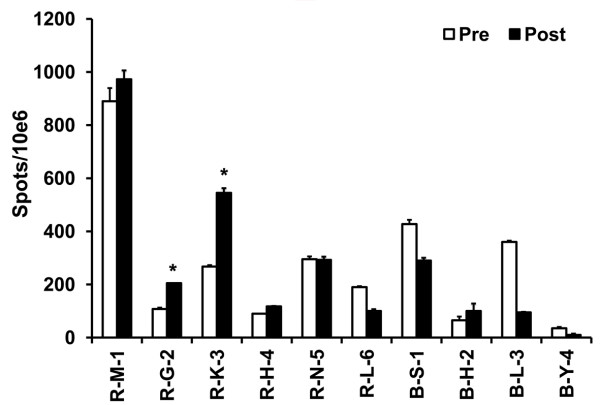
**IFN-γ Secreting T Cells was measured by ELISPOT assay**. ELISPOT assay counting IFN-γ secreting T cells was performed on before (Pre: White bar) and after the vaccination (Post: Black bar). PBMCs were incubated with KLH for 20 h at 37°C to induce the Ag-specific IFN-γ secretion. Stars are indicated the statistical significance (*p < 0.05) comparing before vs. after vaccination.

Frequency of T cells producing IFN-γ that is possible anti-tumor effector was measured by ELISPOT assay with *in vitro *stimulation of KLH. Two (R-G-2, R-K-3) out of ten patients showed significant induction (*P *< 0.05) of IFN-γ-secreting T cells specific to KLH after vaccination (Figure 5). Patient R-G-2 whose disease stabilized in response to vaccination had the highest induction of effect cells specific to KLH. Seven patients whose disease progressed showed no changes (R-M-1, R-N-5) or decreased (R-L-6, B-S-1, B-L-3, B-Y-4) IFN-γ-secreting T cell numbers after DC vaccination.

### Cytokine Responses

**Figure 6:**
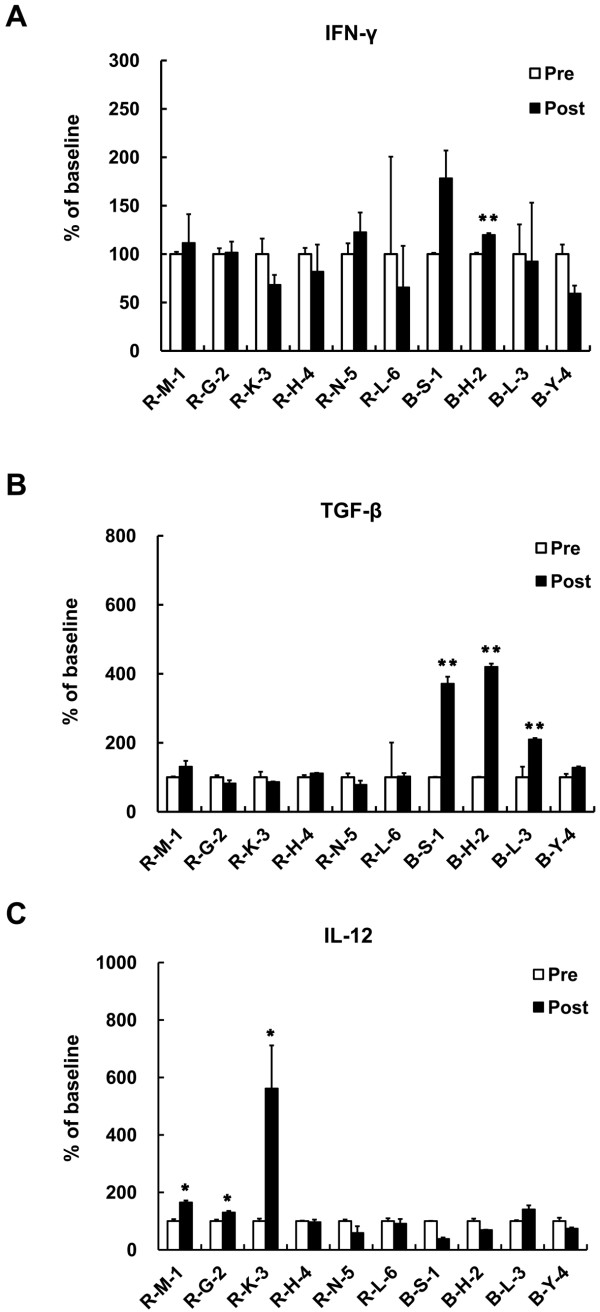
**Cytokine Responses**. DC vaccine induced cytokine (A) IFN-γ (B) TGF-β (C) IL-12 secretion was determined by ELISA. Plasma level of IFN-γ was ranged from 1.31 pg/ml to72.788 pg/ml, of TGF-β was from 41.71 pg/ml to 7305.01 pg/ml, and of IL-12 was from 0.697 pg/ml to 927.789 pg/ml. Data plotted as the relative changes from pre-vaccination baseline. Stars are indicated the statistical significance (*p < 0.05; **p < 0.001) comparing before (Pre: White bar) vs. after vaccination (Post: Black bar).

The plasma concentrations of IL-10 and VEGF did not change significantly (data not shown). As an effector cytokine, induction of IFN-γ level was expected by DC vaccination. However, plasma level of IFN-γ was too low to conclude the relativity to vaccine treatment (Figure 6A). Although the alterations are not statistically significant, level of TGF-β, an immune suppressive cytokine, had a tendency to decrease in patients R-G-2 and R-N-5 after the treatment (from 1028 pg/ml to 847 pg/ml for R-G-2, from 995 pg/ml to 781 pg/ml for R-N-5, respectively) (Figure 6B). The remaining patients showed slightly decreased or not altered TGF-β concentration by vaccination. In two patients (R-M-1, R-G-2), IL-12, a stimulator of Th1 cell-mediated immune responses, was increased by vaccination (from 245.7 pg/ml to 405.8 pg/ml for R-M-1, from 530 pg/ml to 691 pg/ml for R-G-2, respectively) (Figure 6C). Only one patient responded with stabilized disease (R-G-2) showing elevation of IL-12 accompanied by reduction of TGF-β secretion.

## Discussion

In the present study, cancer vaccine-related safety and the immune alteration was reported. The study was phase I/II clinical trial of cancer vaccine composed with autologous HSC-derived DC and IL-2 performed on six RCC and four breast cancer patients. The vaccine was tolerable and induced the antigen-specific immune alterations. (Table 2)

Using DC vaccine as an anti-cancer therapy module has been facilitated by the development of methods to generate DCs from proliferating CD34^+ ^precursors or from non-proliferating CD14^+ ^monocytes. In our study, CD34^+ ^cells cultured with GM-CSF and IFN-γ were expanded and differentiated into the cells with DC characters by about 3000% of initiating HSCs. Cultured-cells secreted IL-12, Th1-response inducing cytokine, more than IL-10 indicates that cultured DCs are possible as a therapeutic-DC (Figure 2).

A number of clinical studies have been carried out since 1994, applying tumor antigen loaded DC based vaccines [2,14,16,22]. In several patients, clinical responses coincided with the induction of specific T-cell responses. Thus, to evaluate efficacy of DC vaccine induction of tumor-specific T cells and the relationship to the clinical response has been demonstrated [6-9]. After vaccination, KLH-specific lymphocyte proliferation was induced in 8 out of 10 patients. However, among KLH-specifically activated immune cells, IFN-γ secreting effector T cell frequency was elevated in only 2 RCC patients (Figure 5). And in one patient of RCC with stable disease, the elevation correlated with clinical outcome. The value of ELISPOT assay measuring effector T cell frequency as a predictor of DC vaccine-related anti-tumor responses was also indicated in other group [23].

In our trial, low-dose IL-2 was injected following DC injection expecting positive influence on the induction of cellular immunity including NK activity. Six out of ten patients showed increasing NK activity after vaccination and in only two patients the increased proportion of CD16^+^CD56^dim ^cytotoxic-NK cell was accompanied by increased activity. However, no relevance to clinical results was observed (Figure 3). As a T cell growth factor, IL-2 was known to induce not only effector T cells but also CD4^+^CD25^+ ^regulatory T cells [19-21]. Although we used low-dose IL-2, increased CD4^+^CD25^+ ^T cell subset proportion was observed in 7 out of 10 patients. Interestingly, the most significant induction of KLH-specific lymphocyte proliferation was indicated in the two patients showing CD4^+^CD25^+ ^T cell subset reduction and clinical response was achieved in this one out of two patients (Figure 4).

High blood levels of TGF-β have been suggested as an indicator of poor prognosis in advanced cancer patients [8,24]. Even though the statistical significant was not observed, in the RCC patient with stabilized disease, reducing phenomenon of TGF-β level was detected after the vaccination. In the same patient, the level of IL-12 was increased by vaccination (Figure 6). Our result could be related to a published report showing association of increased IL-12 level with anti-tumor efficacy in RCC patients receiving immunotherapy [25]. The data suggest that the concomitant alterations of immune inhibitory cytokine (TGF-β) and immune stimulatory cytokine (IL-12) may represent the DC vaccine-induced clinical response.

## Conclusion

Our anti-tumor vaccine with DC and IL-2 was tolerable and induced the antigen-specific cellular immunity in the metastatic RCC and Breast cancer patients. Only the patient with objective clinical response revealed the significant induction of anti-tumor immunity such as IFN-γ producing CD8^+ ^cell proportion and IL-12 secretion at the same time with the reduction of inhibitory immune responses including CD4^+^CD25^+ ^T cell subsets which possibly represent the regulatory T cells and TGF-β secretion. Data suggest that in DC vaccine trial, the objective clinical responses may anticipate when the anti-tumor immunity is stimulated concomitantly with inhibition of regulatory immunity.

## Competing interests

The authors declare that they have no competing interests.

## Authors' contributions

SB carried out the immune monitoring, analyzed the data and wrote the manuscript. CSK, SBK are the clinical protocol PI, performed case collection and treated patients. YK and SWK helped to perform clinical trial. YMK, HSK participated in the design of the study and generated DC vaccine. HL designed the study, supervised immune-monitoring in execution and helped in manuscript revision and discussion. All authors approved the final manuscript.
